# Innovative Integrated Model of Industrial Wastewater Treatment with the Circular Use of Cerium Compounds as Multifunctional Coagulants: Comprehensive Assessment of the Process and Environmental and Economic Aspects

**DOI:** 10.3390/molecules30163428

**Published:** 2025-08-20

**Authors:** Paweł Lejwoda, Barbara Białecka, Anna Śliwińska, Piotr Krawczyk, Maciej Thomas

**Affiliations:** 1 Department of Energy Saving and Air Protection, Central Mining Institute in Katowice, Plac Gwarków 1, 40-166 Katowice, Poland; asliwinska@gig.eu (A.Ś.); pkrawczyk@gig.eu (P.K.); 2 Department of Environmental Monitoring, Central Mining Institute in Katowice, Plac Gwarków 1, 40-166 Katowice, Poland; bbialecka@gig.eu; 3 Faculty of Environmental Engineering and Energy, Cracow University of Technology, Warszawska 24, 31-155 Kraków, Poland

**Keywords:** industrial wastewater treatment, multifunctional coagulants, cerium coagulants, life cycle assessment, rare earth elements

## Abstract

This article presents an innovative method for phosphate(V) removal from industrial wastewater using cerium(III) chloride as a coagulant, integrated with reagent recovery. The process combines coagulation, acid extraction, and multistage recovery of cerium and phosphorus, enabling partial reagent loop closure. Based on our previously published studies, at an optimised dose (81.9 mg Ce^3+^/L), phosphate(V) removal reached 99.86% and total phosphorus (sum of all phosphorus forms as elemental P), 99.56%, and 99.94% of the added cerium was retained in sludge. Reductions were also observed for TSS (96.67%), turbidity (98.18%), and COD (81.86%). The sludge (101.5 g Ce/kg, 22.2 g P/kg) was extracted with HCl, transferring 99.6% of cerium and 97.5% of phosphorus to the solution. Cerium was recovered as cerium(III) oxalate and thermally decomposed to cerium(IV) oxide. Redissolution in HCl and H_2_O_2_ yielded cerium(III) chloride (97.0% recovery and 98.6% purity). The HCl used for extraction can be regenerated on-site from chlorine and hydrogen obtained from gas streams, improving material efficiency. Life cycle assessment (LCA) showed environmental benefits related to eutrophication reduction but burdens from reagent use (notably HCl and oxalic acid). Although costlier than conventional precipitation, this method may suit large-scale applications requiring high phosphorus removal, low sludge, and alignment with circular economy goals.

## 1. Introduction

The Earth’s water resources are estimated at 1.385 billion km^3^, with saline water accounting for 97.5% of the total volume. Freshwater constitutes only 2.5% of the total water resources, which are primarily stored in glaciers and snow cover (approximately 69%), groundwater (30.1%), lakes (0.26%), soil moisture (0.05%), the atmosphere (0.04%), wetlands (0.03%), rivers (0.006%), and biological water (0.003%) [1]. On the one hand, the continuous growth of the global population necessitates addressing the increasing demand for potable water [2]; on the other hand, industrial and domestic human activities contribute to the contamination of water bodies with a wide spectrum of pollutants, including pesticides [3]; heavy metals such as lead (Pb) [4], cadmium (Cd) [5], nickel (Ni) [6], mercury (Hg) [7], copper (Cu) [8], and chromium (Cr) [9]; arsenic compounds (As) [10]; and microplastics [11]. This presents a significant challenge for ensuring high-quality water resources for both current and future generations.

On the other hand, the utilisation of freshwater, depending on the end user, in most cases results in the generation of wastewater with diverse chemical compositions. Domestic wastewater is typically characterised by elevated concentrations of biogenic elements such as carbon (C), nitrogen (N), and phosphorus (P) [12]. Wastewater from the food industry, such as brewery effluents, has a composition similar to domestic wastewater but contains pollutants at higher concentrations. Industrial wastewater, for example from the coking industry [13,14] or coal gasification processes, is characterised by high concentrations of cyanides, phenols, and polycyclic aromatic hydrocarbons (PAHs) [15]. Electroplating wastewater, depending on the type of metal coating applied, may contain elevated levels of heavy metals such as Cu, Ni, Cr, and Cd [16,17].

The increasing physicochemical complexity of wastewater poses a significant challenge to treatment processes. A variety of methods are employed to remove specific groups of contaminants. Metal removal can be achieved, among other methods, by precipitation methods involving the alkalisation of wastewater, followed by the precipitation of metal hydroxides, or by the formation of metal sulphides, according to general reactions (1) and (2) [18].

M^n+^ + nOH^−^ → M(OH)_n_↓ (1)

mM^n+^ + nS^2−^ → M_m_S_n_↓ (2)

However, these methods have several drawbacks, primarily due to their high demand for chemical reagents, significant generation of secondary sludge, and limited effectiveness in the presence of complexed metals (i.e., metals chelated by strong organic ligands such as ethylenediaminetetraacetic acid (EDTA) or natural humic and fulvic acids, commonly found in industrial and municipal wastewater) [19]. Additionally, precipitated sludge often displays low chemical stability. Therefore, to increase metal removal efficiency, various chemical agents, such as sodium dimethyldithiocarbamate (DMDTC) [20], sodium trithiocarbonate (Na_2_CS_3_) [21], trimercapto-s-triazine (TMT) [22], and 1,3-benzenediamidoethanethiol dianion (BDET) [23], are applied. These compounds improve the precipitation performance by forming stable complexes, reducing the metal solubility over a broad pH range, and decreasing the sludge volume.

The removal of substances responsible for turbidity and colour, as well as the reduction in phosphorus concentrations, can be achieved via coagulation using, among other methods, ferric or aluminium salts. These compounds operate through a similar mechanism involving the adsorption of contaminants onto the surface of hydroxide flocs formed during the process [24,25]. The efficiency of coagulants strongly depends on the pH of the treated wastewater, with the optimal pH range for ferric salts being 5.5–7.0 [26] and that for aluminium salts being 5.7–6.0 [27]. In the case of ferric salts, the phosphate(V) removal efficiency may reach 98%, whereas for aluminium salts, it is typically approximately 83%. A major disadvantage of these coagulants is the formation of large volumes of hydrated sludge, which requires further handling. Following treatment, the effluents are usually discharged into receiving bodies, most commonly surface waters such as rivers.

In cases of insufficient wastewater treatment, eutrophication is observed, resulting from excessive enrichment of water with nitrogen and phosphorus, originating mainly from municipal, industrial, and agricultural runoff. It leads to rapid algal growth, increased water turbidity, and oxygen depletion, which can threaten aquatic ecosystems. This phenomenon is also accompanied by the deterioration of water quality in terms of odour and taste [28,29].

The removal of excess nitrogen and phosphorus from water is challenging for conventional wastewater treatment processes, posing a significant challenge for water treatment technologies. The removal of these elements requires advanced biological processes such as denitrification (nitrogen removal) [30] and chemical processes such as coagulation (phosphorus removal) [31]. Given increasing water pollution, it is crucial to implement effective strategies to manage nitrogen and phosphorus emissions in both agricultural and urban areas to prevent further deterioration of water quality [32,33].

Compared with conventional coagulants, more effective removal of pollutants from water and wastewater can be achieved by using innovative coagulants containing zirconium, titanium or, more recently, cerium compounds. For the removal of phosphorus compounds, the use of cerium coagulants seems to be particularly beneficial. As recent reports have shown, the use of cerium(III) chloride in wastewater treatment has been studied in the context of treating leachates from sewage sludge dewatering [34] and brewing wastewater [35]. In the case of brewery wastewater, 99.86% of the phosphates(V) were removed under optimal treatment conditions, indicating high removal efficiency. Furthermore, under optimal conditions, 99.94% of the applied cerium(III) was bound within the sludge. Cerium compounds have proven to be effective not only in removing phosphates(V) but also in removing other contaminants present in industrial wastewater, such as effluents from the hard coal gasification process, where cerium(IV) sulphate (Ce(SO_4_)_2_·4H_2_O) is used for the oxidation of PAHs, cyanides, and phenols [15].

The use of cerium compounds for wastewater treatment is promising; however, rare earth elements (REEs) production is limited to a few countries worldwide (Table 1), posing risks to supply chains in the event of adverse geopolitical developments.

In the European Union, nearly all REEs consumed are imported, and recycling is carried out for only approximately 1% of REEs [37]. Given the higher price of cerium coagulants than of widely used iron and aluminium coagulants, it is important to explore recycling options. Additionally, owing to their initially high effectiveness, further research should be conducted similarly to the previously studied and developed zirconium and titanium coagulants.

Both cerium used as a coagulant and phosphorus removed during the process are classified as critical raw materials for the European Union’s economy. Phosphorus, derived from minerals such as fluorapatite (Ca_5_(PO_4_)_3_F), chlorapatite (Ca_5_(PO_4_)_3_Cl), and hydroxyapatite (Ca_5_(PO_4_)_3_OH), is a key component in the production of mineral fertilisers, which are essential for ensuring efficient agricultural output. Similar to REEs, phosphorus deposits are concentrated in a limited number of countries, including Morocco and China. This creates a significant supply risk and necessitates the search for alternative sources, including recovery from waste streams. Due to the EU’s near-total dependence on imports of these materials, the implementation of technologies enabling the closure of phosphorus and cerium cycles through recovery from wastewater is of particular importance. Integrating such solutions into wastewater treatment systems supports not only the circular economy but also strengthens the raw material autonomy of the European Union [37].

Our studies on cerium recycling from wastewater sludge [38] demonstrated the effective removal of contaminants from industrial wastewater and the feasibility of recovering cerium(III) chloride, cerium(III) sulphate, and cerium(IV) sulphate with efficiencies of 97.0%, 97.4%, and 98.3%, respectively. The recycling process involved cerium extraction from wastewater sludge via hydrochloric acid, the selective precipitation of cerium(III) oxalate, the thermal decomposition of cerium(III) oxalate to cerium(IV) oxide, and the synthesis of salts via hydrogen peroxide, hydrochloric acid, or sulphuric acid. The recovered salts can be reused for wastewater treatment. These studies and experiments on a laboratory and semitechnical scale allowed us to propose a complementary process of recovery and the use of cerium compounds to remove phosphorus from industrial wastewater.

This paper summarises our studies that demonstrate the usefulness of cerium coagulants and their recycling, enabling the closure of the cerium cycle in wastewater treatment [35,38]. The proposed new process for the recovery and use of cerium compounds should be subjected to comprehensive process, environmental, and economic assessments. The literature review revealed that no such comprehensive assessment has been conducted to date. This paper presents the results of our research along with an assessment of the entire upscaled process. The work includes a 10-stage process design, mass balance, LCA evaluation, and cost analysis performed using the DGC method, thus addressing a knowledge gap in this field. Therefore, this work can be an important step in the discussion, research, development, and evaluation of advanced precipitation, coagulation, and recovery processes for better environmental protection and sustainable development.

## 2. Results and Discussion

### 2.1. Process Analysis (Operating Conditions and Initial Assumptions)

The proposed model assumes an influent industrial wastewater flow of 5000 m^3^ per day, containing phosphate(V) at a concentration of 37 mg/L, corresponding to 185 kg of phosphate(V) ions. It is further assumed that the addition of the cerium-based coagulant occurs in the primary settling tanks, with the resulting sludge subjected to a series of processes aimed at recovering the cerium coagulant for reuse in wastewater treatment. On the basis of the optimal coagulant dose described by Lejwoda et al. [35], with a molar ratio of Ce(III) to phosphate(V) of 1.5:1, the required amount of cerium(III) chloride heptahydrate (CeCl_3_·7H_2_O) during start-up is 1088.3 kg, which corresponds to a concentration of 81.9 mg Ce^3+^/L. Assuming a recovery efficiency of 97.1% in the form of CeCl_3_·7H_2_O (1057.3 kg), Stage I will require a top-up dose of approximately 31 kg CeCl_3_·7H_2_O to compensate for losses.

The dewatered sludge obtained in Stage I is assumed to contain approximately 0.62% dry matter, which translates to 4000 kg of dry sludge from 650 m^3^ of wet sludge. The supernatant and the filtrate generated in Stage II were subjected to nitrification. The sludge is transported by a belt conveyor to reactors, each with a volume of 25 m^3^.

In Stage III, 4000 kg of sludge is extracted following the addition of 45,333 kg of water and 4667 kg of 30% hydrochloric acid (HCl). The process requires no heating. The total reaction mass of 54,000 kg is divided into two reactors, with two batches per reactor, resulting in four processing cycles of 13,500 kg each. Each cycle is assumed to last 3 h, after which the reaction mixture is pumped to a filter press.

Stage IV involves filtration of the reaction mass (54,000 kg). The insoluble organic residue (suspended solids) is assumed to be approximately 3406 kg and, following separation, is conveyed for agricultural or energy-related applications.

The extract, weighing approximately 50,600 kg and containing cerium, together with an additional 5000 kg of water used for washing the sludge, is transferred to a buffer tank and then to two 25 m^3^ reactors, where the process is carried out in three consecutive batches per reactor (six batches in total).

Stage V, which includes the precipitation of cerium(III) oxalate (Ce_2_(C_2_O_4_)_3_), begins with the addition of approximately 659 kg of oxalic acid dihydrate (H_2_C_2_O_4_·2H_2_O)—the total required amount for the entire stage. To optimise the precipitation conditions, the pH of the reaction mixture, initially 0.3, is increased to 1.8 by adding approximately 1077 kg of sodium hydroxide (NaOH). The total water content of the NaOH and H_2_C_2_O_4_ solutions was 24,500 kg. The extract contained 3763 mg/L of Ce, and after precipitation, the concentration in the filtrate was reduced to 1.10 mg/L, resulting in a calculated yield of 99.97%.

The process results in the precipitation of 1049 kg of Ce_2_(C_2_O_4_)_3_ (yield: 99.97%). A total reaction mass of 82,281 kg is pumped to a filter press, where Stage VI involves separating the precipitate from the filtrate. Ce_2_(C_2_O_4_)_3_ (approx. 1049 kg) is transferred to a furnace for thermal decomposition. The filtrate (approx. 81,232 kg) was transferred to a storage tank.

The acidic solution (pH 1.8) contains phosphate(V) at a concentration of 570 mg/L, which can be recovered by the addition of calcium hydroxide (Ca(OH)_2_), which also serves as a neutralising agent. Neutralisation and precipitation of calcium phosphate(V) (Ca_3_(PO_4_)_2_) require the addition of 210.32 kg Ca(OH)_2_ per 81,232 kg of filtrate. The resulting suspension is filtered, and the filtrate is pumped to evaporation ponds covering an area of 10 ha.

The thermal decomposition of (Ce_2_(C_2_O_4_)_3_) in Stage VII proceeded with an efficiency of approximately 99.5%, yielding approximately 496.0 kg of cerium(IV) oxide (CeO_2_). The process was conducted over 3 h at a temperature of 350 °C.

Stage VIII involves the synthesis of cerium(III) chloride (CeCl_3_) from the CeO_2_ produced in Stage VII. The oxide is transferred to a 10 m^3^ reactor, where it reacts with HCl and hydrogen peroxide (H_2_O_2_). In the scenario analysed, 496.0 kg of CeO_2_ was subjected to a reaction with 2713.7 kg of 30% HCl and 993.3 kg of 30% H_2_O_2_. The process, carried out at 90 °C for 3 h, achieves a yield of 98.5%, resulting in the production of 1057.3 kg of CeCl_3_. The by-products included approximately 621.1 kg of chlorine gas (Cl_2_) and 7.14 kg of unreacted residue.

Stage IX involves heating the solution to evaporate water, which is then recirculated into earlier stages of the process. At this point, the solution may also be concentrated to reach the desired Ce(III) concentration for reuse in Stage I.

To minimise the environmental impact of by-products and reduce the reagents costs, Stage X was expanded to include the recovery of both chlorine (Cl_2_) and hydrogen (H_2_), enabling the on-site production of HCl and partially eliminating the need for external sourcing. During Stage VIII (CeCl_3_·7H_2_O synthesis), a gaseous by-product containing chlorine (Cl_2_) and oxygen (O_2_) is generated. This mixture is separated via a membrane system, which recovers approximately 619 kg/day of pure Cl_2_. The chlorine is then directed to an HCl synthesis reactor, where it reacts with hydrogen.

Hydrogen is derived from sewage sludge, which is first subjected to anaerobic digestion, producing biogas (containing methane (CH_4)_ and carbon dioxide (CO_2_)) and digested sludge. The biogas may be utilised for in-house energy generation, reducing reliance on external suppliers. The digested sludge is then gasified to yield synthesis gas containing H_2_ (approx. 19.3 kg/day), carbon monoxide (CO), and carbon dioxide (CO_2_). Using a membrane separation module, H_2_ is isolated and fed into the HCl synthesis reactor. The resulting HCl gas is absorbed in water within an absorption column to produce a 30% HCl solution. This solution (~1819 kg/day) can then be reused in Stage III for cerium extraction from sludge, effectively closing the reagent loop. Hydrochloric acid constitutes a significant portion of the chemicals used in the process. Implementing recovery reduces the demand for external supply by approximately 24.6%. The implementation of this modification significantly enhances the material and energy efficiency of the system while reducing CO_2_ and Cl_2_ emissions to the atmosphere. Additionally, surplus hydrogen may be utilised as a fuel for the plant’s energy needs or sold externally. The use of this hydrochloric acid recovery technology aligns with the principles of the circular economy, increasing the operational independence of the facility and minimising the environmental footprint of the entire wastewater treatment process.

**Figure 1:**
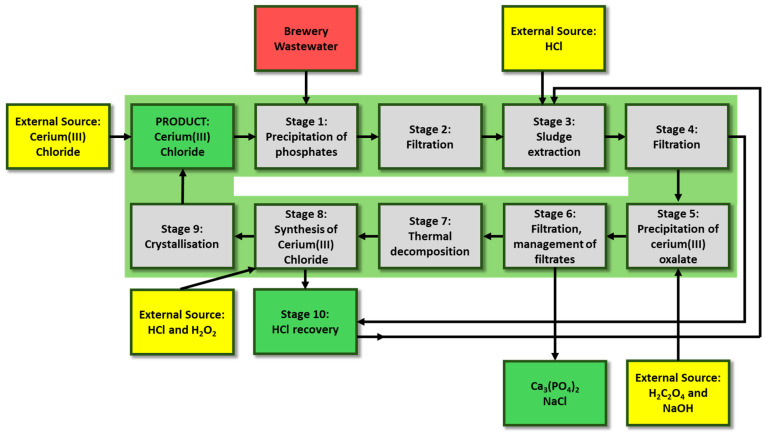
Conceptual diagram of wastewater treatment with cerium coagulant recirculation.

The integration of all the aforementioned processes enables the formation of a nearly closed-loop system for cerium in wastewater treatment (Figure 1), forming the basis for further process, environmental, and economic evaluation of the proposed technology.

The developed concept comprises ten stages (Table 2), aimed at achieving near-complete closure of the cerium loop within the treatment system.

### 2.2. Environmental Assessment

Table 3 presents the environmental impact results over the life cycle of the wastewater treatment process with phosphate(V) recovery.

**Figure 2:**
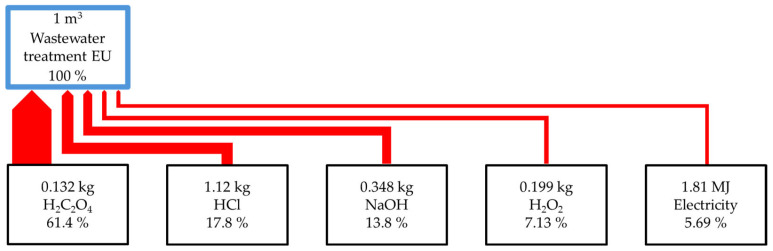
Carbon footprint.

The life cycle assessment (LCA) conducted via the ReCiPe 2016 method allows for the evaluation of various process stages and materials used in relation to their environmental impact. The analysis considers impact categories such as climate change, ozone layer depletion, acidification, eutrophication, toxicity, and resource consumption. The results indicate that H_2_C_2_O_4_ and HCl have the greatest impact on multiple categories. Oxalic acid is the dominant contributor to climate change (2.04 kg CO_2_-eq), terrestrial ecotoxicity (5.96 kg 1.4-DCB-eq), and fossil resource depletion (0.479 kg oil-equivalent). Hydrochloric acid significantly affects terrestrial ecotoxicity (5.41 kg 1,4-DCB-eq) and marine ecotoxicity (7.85 × 10^−2^ kg 1.4-DCB-eq). Oxalic acid and hydrochloric acid along with hydrogen peroxide are main contributors which impact human carcinogenic toxicity. Improving environmental efficiency could be achieved through enhancing the production efficiency of oxalic acid and hydrochloric acid, replacing these substances with alternatives from sustainable sources, and considering less harmful substitutes whenever possible. Additionally, energy consumption at various process stages is a significant source of environmental impact, highlighting the need to improve energy efficiency and transition to renewable energy sources. Figure 2 shows the carbon footprint of the analysed process.

The results indicate that the greatest climate impact is associated with the production of oxalic acid (indirect emission), the calcination of oxalic acid (direct emission), and the consumption of hydrochloric acid.

**Figure 3:**
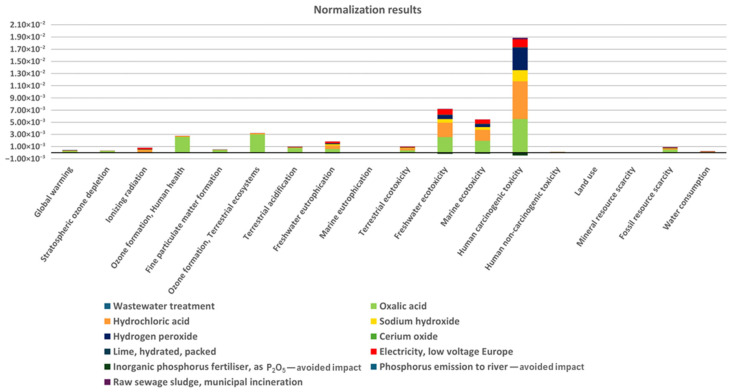
Normalised environmental impact results via the ReCiPe 2016 midpoint (H/A) method.

In the next phase, the normalised results were examined. Normalisation in LCA involves transforming environmental impact results into comparable values by referencing a defined benchmark (e.g., the total environmental impact of a specific region or population). This approach helps determine which impact categories are most significant. Figure 3 presents the normalised environmental impact results calculated via the ReCiPe 2016 midpoint (H/A) method. Normalisation in this method was performed using global per capita reference values derived from annual emissions and resource use data for the year 2010, enabling relative comparison of environmental impacts [41].

The highest environmental burden is observed in the category of human noncarcinogenic toxicity, with oxalic acid being the dominant contributor. The next two dominant categories are freshwater ecotoxicity and marine ecotoxicity, and this impact is also associated with the production of oxalic acid, hydrochloric acid, and sodium hydroxide. Although greenhouse gas emissions and resource consumption are important concerns, they are not the dominant issues in the analysed system.

The goal of the analysed process is the recovery of phosphate(V) (PO_4_^3−^) from wastewater, which is one of the main factors causing eutrophication, i.e., the enrichment of aquatic ecosystems with excessive amounts of nutrients, leading to uncontrolled algal growth, oxygen depletion in water, the death of aquatic organisms, the proliferation of toxic cyanobacteria species, and the disruption of aquatic ecosystem balance.

By effectively treating wastewater and reducing phosphate(V) emissions into rivers and lakes, the process significantly reduces the impact in the eutrophication-related category. In the freshwater eutrophication impact category, a negative value (impact reduction) appears, indicating that avoiding phosphate(V) emissions significantly reduces eutrophication. A similar situation occurs in marine eutrophication, where reducing phosphorus emissions lowers the negative impact on coastal ecosystems. Furthermore, phosphorus recovery from wastewater sludge and the reuse of the obtained calcium phosphate(V) as a substitute for phosphate(V) fertilisers reduce the need for phosphate(V) resource exploitation and subsequent environmental emissions. The proposed phosphate(V) emission minimisation technology plays a significant role in protecting freshwater bodies and marine ecosystems from degradation.

### 2.3. Economic Assessment

Table 4 presents the calculation results for the selected years covered in the economic analysis, which were used to determine the DGC indicator.

The economic analysis conducted via the dynamic generation cost (DGC) method revealed that the cost of phosphate (V) removal from wastewater via cerium(III) chloride and its recovery is 1.37 USD/m^3^. As of Q1 2025, the average net wastewater fee calculated for 97 major cities in Poland [42] was approximately 2.13 USD/m^3^. This cost includes not only wastewater treatment but also the transportation of wastewater to treatment plants via the sewage system and the general operational costs of enterprises managing urban wastewater systems. At municipal wastewater treatment plants that use standard phosphate(V) removal methods (typically chemical precipitation), the cost of phosphate(V) removal represents a small fraction of the overall operational cost of the facility. However, precise data on what proportion of the total expenses it constitutes are not readily available.

A review of market prices and literature sources [34,43] indicates that cerium(III) chloride prices can be 2.5 to 14 times higher than those of commonly used iron- and aluminium-based coagulants. Consequently, technology based on cerium(III) chloride and its recovery may be significantly more expensive than simpler chemical precipitation methods. However, the use of cerium-based coagulants reduces the amount of wastewater sludge generated by approximately 20–25% compared with traditional methods. For large wastewater treatment plants, lower sludge production translates into significantly reduced costs associated with its management and disposal. Additionally, the phosphate(V) removal efficiency of over 99.8% achieved with this technology is practically unattainable via conventional chemical precipitation methods. Ultimately, despite the higher purchase costs of the coagulant, this method may be economically justified in wastewater treatment plants where high phosphate(V) removal efficiency and minimised sludge management costs are a priority.

## 3. Materials and Methods

### 3.1. Assumptions Underlying the Design of a Cerium-Based Wastewater Treatment Process

On the basis of the results of our previous research on the recovery, synthesis, properties, and application of cerium compounds [15,35,38], which were conducted at both laboratory and semitechnical scales, an innovative solution for industrial wastewater treatment using cerium(III) chloride heptahydrate (CeCl_3_·7H_2_O) as a coagulant has been proposed. Although this concept was developed specifically for brewery wastewater, it may also be suitable for other types of industrial effluents containing phosphorus compounds. Furthermore, it is assumed that the proposed solution can be applied wherever efficient precipitation of phosphorus from contaminated water or wastewater is particularly important. The main objective of this process is the cyclic recovery of cerium from sewage sludge and the management of by-products generated during recycling.

Stage I involves the removal of phosphate(V) from brewery wastewater through the precipitation of poorly soluble cerium(III) phosphate(V), according to reaction (3) [34,44,45,46]. A molar ratio of Ce:PO_4_^3−^ equal to 1.5:1 was adopted for calculations on the basis of the study by Lejwoda et al. [35], which ensures efficient phosphate(V) removal with a minimal residual concentration of cerium coagulant in the treated effluent.

Ce^3+^ + PO_4_^3−^ → CePO_4_↓ (3)

Stage II comprises sedimentation and filtration of the resulting sludge. The sludge obtained contains, on average, 10.15 wt% of cerium and 2.22 wt% of phosphorus, making it a valuable material for recovery. In Stage III, cerium and phosphorus are extracted from wastewater sludge via hydrochloric acid, following reaction (4) [38].

CePO_4_↓ + 3HCl + 3H_2_O → Ce^3+^ + PO_4_^3−^ + 3H_3_O^+^ + 3Cl^−^ (4)

The solubilisation of cerium and phosphorus enables, in Stage IV, the separation of dissolved substances from undissolved organic matter, which is subsequently used as feedstock for the processes described in Stage X.

In Stage V, oxalic acid is first added to the acidic extract (pH 0.3), followed by pH adjustment to 1.8 using sodium hydroxide (NaOH). The selective action of oxalic acid under these conditions leads to the precipitation of poorly soluble cerium(III) oxalate, as shown in reaction (5) [47,48,49].

2Ce^3+^ + 3C_2_O_4_^2−^ + nH_2_O→ Ce_2_(C_2_O_4_)_3_·nH_2_O↓ (where n = 9, 10) (5)

Stage VI involves the filtration of the resulting suspension, with the solid cerium(III) oxalate directed to Stage VII, while the acidic filtrate is neutralised, and the phosphate(V) content is removed via precipitation with calcium hydroxide (Ca(OH)_2_). The resulting solid is filtered, and the brine is evaporated in solar ponds to recover NaCl.

Stage VII is the thermal decomposition of cerium(III) oxalate at 350 °C to obtain fine-crystalline CeO_2_ according to reaction (6), which is more reactive in subsequent processes than the cerium(IV) oxide produced at higher temperatures [38,50,51].

Ce_2_(C_2_O_4_)_3_·nH_2_O + 2O_2_→ 2CeO_2_ + 6CO_2_ + nH_2_O (where n = 9, 10) (6)

Stage VIII consists of the synthesis of cerium(III) chloride. This is achieved by reducing Ce(IV) to Ce(III) via the use of hydrogen peroxide (30 wt%) in hydrochloric acid (30 wt%) applied in 100% and 200% excess. This allows the conversion of cerium(IV) to cerium(III), followed by the formation of cerium(III) chloride through concentration and crystallization, according to reactions (7) and (8) [38,52].

Ce^4+^ + e^−^ → Ce^3+^ (7)

Ce^3+^ + 3Cl^−^ → CeCl_3_ (8)

Depending on the scale of demand, Stage IX may include the concentration of the CeCl_3_ solution to the level required in Stage I or complete crystallisation, with the evaporated water being redirected to earlier stages of the process.

To assess the process comprehensively, Stage X is introduced, with a focus on minimising waste streams. The gaseous by-products of Stage VIII (O_2_ and Cl_2_) are separated via a membrane system [53]. Chlorine can be used for HCl synthesis via reaction with hydrogen (reaction 9), which is obtained through the gasification of organic matter remaining after Stage III.

Fermented and/or gasified sludge may also serve as a source of methane (CH_4_) and carbon monoxide (CO), which can be utilised for energy production within the facility.

H_2_ + Cl_2_ → 2HCl (9)

The concentrations of elements at individual stages of the process (our studies are marked in Table 2) were obtained from laboratory analyses performed using, among others, ICP-OES and ICP-MS techniques, in accordance with EN ISO standards and our own laboratory procedures, covering a total of 50 elements: Ca, Mg, Na, K, Li, Ba, Sr, Fe, Mn, Cd, Be, Ti, Cr, Co, Zr, V, Cu, Mo, Ni, Pb, B, Zn, Al, Sb, Ag, As, Se, Sn, Bi, S, Si, P, Tl, Hg, Te, Ce, Dy, Er, Eu, Gd, Ho, La, Lu, Nd, Pr, Sm, Tb, Th, Tm, Y, Yb, and U. Measurement uncertainties ranged from 10% to 25%. The concentrations of the vast majority of these elements were below 0.005 mg/L or 0.001 mg/L (for ICP-OES) and 0.05 µg/L or 0.01 µg/L (for ICP-MS); therefore, during the development of the laboratory-scale process, they were omitted as irrelevant from a technical and technological perspective. The analyses were conducted in an accredited laboratory (Central Mining Institute—National Research Institute in Katowice, Poland).

### 3.2. Process Analysis (Operating Conditions and Initial Assumptions)

A process analysis was conducted as a preliminary assessment of the technological feasibility of the proposed wastewater treatment process employing a cerium-based coagulant and its subsequent recycling. The analysis incorporated realistic operational conditions by scaling the results of previous laboratory- and pilot-scale studies to the conditions of a small wastewater treatment plant with a daily capacity of 5000 m^3^. Initial process assumptions, including pollutant concentrations and reagent doses and concentrations, as well as the efficiencies of individual process stages, were adopted.

Each of the ten stages of the process was examined in terms of reaction implementation, technological parameters, equipment configuration (e.g., reactors, filters, filter presses, and buffer tanks), and the mass balance of reagents and products. The main and by-products—including sludge, filtrates, and gaseous reaction products—were identified and quantified, particularly with respect to their potential environmental impact. The possibility of managing waste streams through hydrochloric acid recovery, the use of residual sludge as an energy source, and the recovery of salts from postreaction waters was assumed, with the aim of minimising pollutant emissions to the environment.

The process analysis was intended not only to confirm the technological coherence and effectiveness of the proposed solution but also to identify potential critical points that could affect environmental performance and operational costs. The results of the analysis were then used for further environmental assessment via the life cycle assessment (LCA) methodology and for economic evaluation via the dynamic generation cost (DGC) indicator.

### 3.3. Environmental Assessment

The environmental assessment was conducted via the life cycle assessment (LCA) method, following ISO 14040 [54] and ISO 14044 [55] in four phases: (1) goal and scope definition, (2) inventory analysis (LCI), (3) impact assessment (LCIA), and (4) interpretation. Life cycle assessment (LCA) is a method for identifying potential environmental risks associated with the production, use, and disposal of a product. It evaluates the environmental impact of a product by quantifying raw material and energy consumption as well as emissions of waste and gases into the environment. A key feature of this approach is that it covers the entire product life cycle—from raw material extraction, transport, and processing through manufacturing processes, usage, and, finally, waste management or reuse. The advantage of LCA is its comprehensive approach, which considers all environmental impact factors as well as various ecological mechanisms and environmental issues [56]. An LCA supports decision-making processes, enabling the evaluation of existing processes and products as well as the design of new technological solutions [57]. The greatest environmental and economic benefits can be achieved by reducing the environmental impact at the early stages of production, which is the main goal of eco-design [58].

The purpose of this analysis was to assess the environmental impact associated with operations, material consumption, emissions, and waste generation resulting from the recovery of phosphates(V) from wastewater streams. The functional unit was defined as 1,825,000 m^3^ of wastewater treated per year, with all results normalised per 1 m^3^ of wastewater. The ReCiPe 2016 method was applied for impact assessment [41]. ReCiPe 2016 enables the assessment of potential environmental impacts in seventeen various impact categories (midpoints): climate change, ozone depletion, ionising radiation, fine particulate matter formation, and photochemical oxidant formation: terrestrial ecosystems, human health, terrestrial acidification, and freshwater eutrophication; human toxicity: cancer, noncancer, terrestrial ecotoxicity, freshwater ecotoxicity, and marine ecotoxicity; land use, water use, mineral resource scarcity and fossil resource scarcity. The hierarchist perspective was used during calculations, assuming a compromise between long-term and short-term environmental effects.

**Figure 4:**
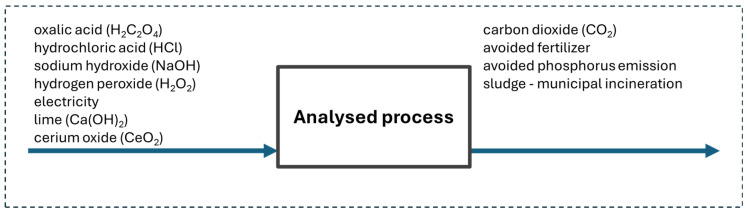
System boundaries.

The system boundaries included the life cycle associated with the production of materials (chemicals) and electricity, as well as the generated wastewater sludge. The analysed system performs two main functions: wastewater treatment and phosphorus recovery. The multifunctionality issue was addressed by expanding the system boundaries—considering the avoided environmental impact associated with the production of phosphate(V) fertilisers. It was assumed that wastewater sludge is neutralised in a waste incineration plant. The system boundaries included in the analysis are illustrated in Figure 4.

### 3.4. Economic Assessment Methodology

Since the analysed technology for phosphate(V) removal using cerium(III) chloride complements the comprehensive wastewater treatment process, it is not possible to charge separate fees for its implementation in treatment plants. Therefore, an economic assessment was conducted via the dynamic generation cost (DGC) method. The dynamic unit cost is equal to the price that allows for discounted revenues to equal discounted costs. DGC demonstrates the technical cost of obtaining a unit of the product (in this project, 1 m^3^ of treated wastewater). Since it does not consider revenues but includes investment and operational costs (product life cycle), its value reflects the unit life cycle cost of the analysed product or technology. When comparing DGC indicators calculated for different technologies, lower values are considered more favourable. The DGC indicator is calculated via the following formula [59]:

$$DGC=\;\frac{\sum_{t=0}^{n}\frac{{KI}_{t}+{KE}_{t}}{{\left(1+i\right)}^{t}}}{\sum_{t=0}^{n}\frac{P_{t}}{{\left(1+i\right)}^{t}}}$$

where

*KI_t_*—Investment costs incurred in a given year;

*KE_t_*—Operational costs incurred in a given year;

*P_t_*—Volume of treated wastewater in a given year;

*i*—Discount rate;

*t*—Year, ranging from 0 to n, where 0 is the year when the first costs are incurred, and n is the final year of wastewater treatment plant operation.

Investment expenditures were estimated on the basis of the proposed installation concept for phosphate(V) removal via cerium(III) chloride presented in this article, as well as the available online price lists for equipment and technological components. A summary of investment expenditures (price level: Q1 2025) is presented in Table 5.

Similar to the investment expenditures, the operational costs were estimated on the basis of the proposed installation concept for phosphate(V) removal via cerium(III) chloride, as presented in this article, and available online price lists for chemicals used in the analysed wastewater treatment process. A summary of the operational costs (price level: Q1 2025) is presented in Table 6.

Recommended Practice 18R-97 of the Association for the Advancement of Cost Engineering International (AACE) describes a Cost Estimate Classification System as applied in Engineering, Procurement, and Construction for the process industries [60]. The techno-economic studies conducted in this article provide cost estimates at the “Study or Feasibility” level (AACE Class 4). Cost estimates in the Class 4 have an expected accuracy range of −15% to −30% on the low side and +20% to +50% on the high side.

For the purpose of dynamic generation cost (DGC) analysis of the developed phosphate(V) removal technology, a discount rate of 4% was applied. This value is recommended in the guidelines [61] for financial analyses conducted at constant prices. The analysis covers a 22-year period (2025–2046), including a 2-year investment implementation period and 20 years of plant operation. Throughout this period, the operational costs of the analysed phosphate(V) removal installation were adjusted via the macroeconomic indices presented in the guidelines [62] and the energy price forecasts outlined in the study [63].

## 4. Conclusions

The wastewater treatment process employing cerium(III) chloride was shown in our previous studies to reduce phosphate(V) concentration by more than 99.86%. The coagulant can also be recovered via acid extraction using hydrochloric acid, with a recovery efficiency of 97.0–98.3%. This enables the reuse of the recovered salt in wastewater treatment processes, with only minimal replenishment required to compensate for losses. An additional advantage of this process is the utilisation of waste materials for energy production or as substrates for the chemical manufacturing required by the treatment plant. The closed-loop reagent cycle in both wastewater treatment and cerium(III) recycling ensures that this innovative technology aligns with the principles of the circular economy.

The conducted life cycle assessment (LCA) demonstrated that the proposed phosphate(V) emission minimisation technology plays a significant role in protecting freshwater bodies and marine ecosystems from degradation. Moreover, the proposed process has adverse environmental impacts, primarily due to the consumption of chemical reagents, notably oxalic acid and hydrochloric acid. The most critical environmental concern identified within the analysed system is related to human carcinogenic toxicity.

The economic analysis revealed that this technology is more expensive than commonly used phosphate(V) removal methods, which are based on chemical precipitation. However, in situations where significantly higher phosphate(V) removal efficiency is needed, the use of cerium(III) chloride becomes an economically justified alternative. The combination of environmental and economic analysis results with the excellent outcomes of experimental studies suggests that this promising technology is worthy of further development and modification to further reduce its environmental impact, lower its operating costs, and improve the utilisation of process by-products.

A recommendation for future research is to compare the efficiency, as well as the environmental and economic aspects, of the proposed solution with other innovative coagulants, such as those based on zirconium or titanium salts, as well as with conventional methods using iron and aluminium salts.

## Figures and Tables

**Table 1:** Contribution of individual producers to REE production and reserves [36].

Country	REEs Production in 2024 (Tonnes)	REEs Reserves (Tonnes)
China	270,000	44,000,000
USA	45,000	1,900,000
Myanmar	31,000	Not available
Thailand	13,000	4500
Nigeria	13,000	Not available
Australia	13,000	5,700,000
Russia	2500	3,800,000
India	2900	6,900,000
Madagascar	2000	Not available
Vietnam	300	3,500,000
Brazil	20	21,000,000
Malaysia	130	Not available
Canada	Not available	830,000
South Africa	Not available	860,000
Greenland	Not available	1,500,000
Tanzania	Not available	890,000
Other countries	1100	Not available

**Table 2:** Subsequent stages of the use and recovery of cerium compounds.

Stage No.	Stage
I	Precipitation of phosphates(V) (a)
II	Filtration (a)
III	Sludge extraction (a)
IV	Filtration (a)
V	Precipitation of cerium(III) oxalate (a)
VI	Filtration, management of filtrates (a,b)
VII	Thermal decomposition (a)
VIII	Synthesis of cerium(III) chloride (a,b)
IX	Crystallisation (a)
X	HCl recovery (b)

a—results from our previous studies [35,38]. b—results from studies conducted by other authors [39,40].

**Table 3:** Results of characterisation—ReCiPe2016 midpoint H.

Impact Category	Unit	Total	1	2	3	4	5	6	7	8	9	10	11
Global warming	kg CO_2_ eq	3.30	2.08 × 10^−4^	2.04	5.80 × 10^−1^	2.84 × 10^−1^	2.35 × 10^−1^	6.85 × 10^−3^	3.91 × 10^−2^	1.88 × 10^−1^	−7.86 × 10^−2^	0.00	7.67 × 10^−3^
Stratospheric ozone depletion	kg CFC11 eq	1.92 × 10^−5^	0.00	1.80 × 10^−5^	7.94 × 10^−7^	2.86 × 10^−7^	5.75 × 10^−8^	3.83 × 10^−9^	1.32 × 10^−9^	8.65 × 10^−8^	−1.02 × 10^−7^	0.00	7.56 × 10^−8^
Ionising radiation	kBq Co60 eq	3.75 × 10^−1^	0.00	3.93 × 10^−2^	1.68 × 10^−1^	3.16 × 10^−2^	3.28 × 10^−2^	3.42 × 10^−4^	3.89 × 10^−4^	1.08 × 10^−1^	−6.13 × 10^−3^	0.00	7.91 × 10^−4^
Ozone formation, Human health	kg NOx eq	5.64 × 10^−2^	0.00	5.36 × 10^−2^	1.30 × 10^−3^	7.58 × 10^−4^	3.84 × 10^−4^	2.71 × 10^−5^	2.69 × 10^−5^	3.48 × 10^−4^	−1.54 × 10^−4^	0.00	9.61 × 10^−5^
Fine particulate matter formation	kg PM2.5 eq	1.25 × 10^−2^	0.00	1.02 × 10^−2^	1.39 × 10^−3^	5.80 × 10^−4^	2.12 × 10^−4^	1.35 × 10^−5^	8.42 × 10^−6^	2.88 × 10^−4^	−1.94 × 10^−4^	0.00	1.67 × 10^−5^
Ozone formation, Terrestrial ecosystems	kg NOx eq	5.67 × 10^−2^	0.00	5.38 × 10^−2^	1.36 × 10^−3^	7.75 × 10^−4^	4.22 × 10^−4^	2.76 × 10^−5^	3.02 × 10^−5^	3.60 × 10^−4^	−1.64 × 10^−4^	0.00	9.75 × 10^−5^
Terrestrial acidification	kg SO2 eq	3.77 × 10^−2^	0.00	3.21 × 10^−2^	3.89 × 10^−3^	1.04 × 10^−3^	5.59 × 10^−4^	2.83 × 10^−5^	2.28 × 10^−5^	7.32 × 10^−4^	−6.67 × 10^−4^	0.00	4.88 × 10^−5^
Freshwater eutrophication	kg P eq	1.15 × 10^−3^	0.00	3.94 × 10^−4^	3.79 × 10^−4^	1.33 × 10^−4^	8.81 × 10^−5^	3.22 × 10^−6^	6.27 × 10^−7^	1.75 × 10^−4^	−2.78 × 10^−5^	−3.31 × 10^−5^	3.57 × 10^−5^
Marine eutrophication	kg N eq	2.48 × 10^−4^	0.00	2.74 × 10^−5^	5.53 × 10^−5^	1.19 × 10^−5^	1.54 × 10^−5^	2.29 × 10^−5^	3.29 × 10^−7^	1.25 × 10^−5^	−3.30 × 10^−6^	0.00	1.05 × 10^−4^
Terrestrial ecotoxicity	kg 1,4-DCB	1.42 × 10^1^	0.00	5.96	5.41	1.25	9.31 × 10^−1^	4.96 × 10^−2^	1.45 × 10^−2^	1.09	−5.30 × 10^−1^	0.00	2.40 × 10^−2^
Freshwater ecotoxicity	kg 1,4-DCB	1.77 × 10^−1^	0.00	6.43 × 10^−2^	6.00 × 10^−2^	1.49 × 10^−2^	1.65 × 10^−2^	1.18 × 10^−3^	6.60 × 10^−5^	2.32 × 10^−2^	−4.79 × 10^−3^	0.00	1.65 × 10^−3^
Marine ecotoxicity	kg 1,4-DCB	2.32 × 10^−1^	0.00	8.50 × 10^−2^	7.85 × 10^−2^	1.96 × 10^−2^	2.18 × 10^−2^	1.52 × 10^−3^	1.00 × 10^−4^	2.94 × 10^−2^	−6.32 × 10^−3^	0.00	2.03 × 10^−3^
Human carcinogenic toxicity	kg 1,4-DCB	1.90 × 10^−1^	0.00	5.68 × 10^−2^	6.40 × 10^−2^	1.87 × 10^−2^	3.82 × 10^−2^	4.92 × 10^−4^	1.70 × 10^−4^	1.33 × 10^−2^	−4.70 × 10^−3^	0.00	2.72 × 10^−3^
Human noncarcinogenic toxicity	kg 1,4-DCB	3.65	0.00	1.49	1.18	3.31 × 10^−1^	3.30 × 10^−1^	4.05 × 10^−2^	1.70 × 10^−3^	3.54 × 10^−1^	−9.10 × 10^−2^	0.00	1.44 × 10^−2^
Land use	m2a crop eq	5.22 × 10^−2^	0.00	1.82 × 10^−2^	2.11 × 10^−2^	6.82 × 10^−3^	4.41 × 10^−3^	6.49 × 10^−4^	5.39 × 10^−4^	6.17 × 10^−3^	−5.83 × 10^−3^	0.00	1.10 × 10^−4^
Mineral resource scarcity	kg Cu eq	1.01 × 10^−2^	0.00	4.16 × 10^−3^	4.32 × 10^−3^	9.88 × 10^−4^	9.33 × 10^−4^	1.08 × 10^−3^	7.84 × 10^−6^	7.31 × 10^−4^	−2.15 × 10^−3^	0.00	3.61 × 10^−5^
Fossil resource scarcity	kg oil eq	8.40 × 10^−1^	0.00	4.79 × 10^−1^	1.79 × 10^−1^	6.90 × 10^−2^	7.92 × 10^−2^	1.55 × 10^−3^	3.55 × 10^−3^	5.09 × 10^−2^	−2.28 × 10^−2^	0.00	1.20 × 10^−3^
Water consumption	m3	5.87 × 10^−2^	0.00	1.55 × 10^−2^	1.92 × 10^−2^	6.76 × 10^−3^	1.51 × 10^−2^	−8.24 × 10^−6^	5.30 × 10^−5^	3.29 × 10^−3^	−1.38 × 10^−3^	0.00	1.40 × 10^−4^

(1) Wastewater treatment; (2) Oxalic acid, H_2_C_2_O_4_; (3) Hydrochloric acid, HCl; (4) Sodium hydroxide, NaOH; (5) Hydrogen peroxide, H_2_O_2_; (6) Cerium(IV) oxide, CeO_2_; (7) Lime, hydrated, packed, Ca(OH)_2_; (8) Electricity; (9) Inorganic phosphorus fertiliser, such as P_2_O_5_, prevents impact; (10) Phosphorus emission to river—avoided impact; (11) Raw sewage sludge, municipal incineration.

**Table 4:** Calculation results for selected years of the economic analysis used for DGC calculation.

Item	Unit	Construction Period		Plant Operation Period				
		2025	2026	2027	2030	2035	2040	2046
Investment expenditures	USD	1,202,530	1,202,530	0	0	0	0	0
Raw materials, chemicals	USD	0	0	1,700,228	1,846,185	1,916,928	1,935,208	1,952,690
Maintenance and repairs	USD	0	0	16,941	18,396	19,101	19,283	19,457
Electricity	USD	0	0	263,372	287,793	260,142	235,148	208,304
Employee salaries	USD	0	0	114,133	123,268	139,331	156,566	179,454
Monitoring	USD	0	0	19,324	20,983	21,787	21,995	22,193
Total—Costs	USD	1,202,530	1,202,530	2,113,997	2,296,625	2,357,288	2,368,200	2,382,098
Volume of treated wastewater	m^3^/year	0	0	1,825,000	1,825,000	1,825,000	1,825,000	1,825,000
Discount factor	-	1.00000	0.96154	0.92456	0.82193	0.67556	0.55526	0.43883
Discounted costs	USD	1,202,530	1,156,279	1,954,509	1,887,658	1,592,500	1,314,977	1,045,345
Discounted volume of wastewater	m^3^/year	0	0	1,687,315	1,500,017	1,232,905	1,013,358	800,871
Total discounted costs	USD	32,670,246						
Total discounted volume of wastewater	m^3^	23,848,409						

**Table 5:** Summary of investment expenditures for the implementation of a phosphate(V) removal installation using cerium(III) chloride with a capacity of 5000 m^3^/day.

Item	Cost [USD]
Stage I. Phosphate(V) precipitation	
Coagulant preparation tank with mixing	5000
Coagulant dosing pump	4000
Wastewater tank for phosphate(V) removal	50,000
Pump for wastewater discharge into the nitrification process (87% of wastewater)	2000
Pump for sludge discharge to filter press (650 m^3^)	2100
Stage II. Filtration	
Filter press × 2	12,000
Pump for filtrate discharge into the nitrification process	2100
Belt conveyor to reactors × 2	2000
Stage III. Sludge extraction	
Acid-resistant reactor for extraction × 2	76,000
30% HCl storage tank for extraction	5000
Acid-resistant pump for HCl	4000
Water storage tank	5000
Water pump	1500
Acid-resistant pump for acidic extract	8000
Stage IV. Filtration	
Acid-resistant filter press	12,000
Belt conveyor for sludge transport for agricultural use	1000
Acid-resistant pump for acidic extract	4000
Acid-resistant reactor for cerium(III) oxalate precipitation × 2	76,000
Stage V. Cerium(III) oxalate precipitation	
NaOH preparation tank with mixing	8000
Alkali-resistant pump	4000
Oxalic acid preparation tank	8000
Acid-resistant pump × 2	8000
Stage VI. Filtration and filtrate management	
Acid-resistant filter press	6000
Belt conveyor for cerium oxalate transport	2000
Acid-resistant pump	8000
Reaction tank with agitator	130,000
Sedimentation tank	70,000
Filter press	75,000
Lime dosing system	30,000
Land preparation, membrane, drainage systems, and barriers	600,000
Stage VII. Thermal decomposition	
Furnace for cerium(III) oxalate decomposition into cerium(IV) oxide at 350 °C	5500
Belt conveyor for cerium(IV) oxide to CeCl_3_ synthesis reactor	1000
Stage VIII. CeCl_3_·7H_2_O synthesis	
Acid-resistant reactor	20,000
Acid-resistant pump for HCl	4000
H_2_O_2_ storage tank	5400
High-resistance pump for H_2_O_2_	4000
Acid-resistant pump	4000
Stage IX. Crystallisation	
Evaporator for concentration	350,000
Coagulant dosing pump—recirculation after recovery	4000
Stage X. Hydrochloric acid recovery	
Gasification reactor, control system, and pumps	60,000
Membrane module	15,000
High-pressure tanks, compressor	12,260
Tubular reactor, cooling, and control system	82,500
Absorption tower and pumps	55,000
Membrane module for Cl_2_ separation	5700
Pressure tank for Cl_2_	12,000
Pumps, pipelines, and process control system	25,000
Total—Stages I to X	1,886,060
Additional equipment + piping	200,000
Automation and control system	150,000
Buildings	75,000
Project documentation, administrative decisions, and permits	94,000
Total—CAPEX	2,405,060

**Table 6:** Summary of operational costs for phosphate(V) removal installation using cerium(III) chloride with a capacity of 5000 m^3^/day.

Item	Unit	Amount	Cost [USD/Year]
Electricity	kWh	993,006	248,253
Monitoring	-	-	18,250
Labour	-	-	108,000
Maintenance and repairs	-	-	16,000
CeCl_3_·7H_2_O	kg/year	11,315	16,973
30% HCl solution	kg/year	2,693,956	1,131,461
30% H_2_O_2_ solution	kg/year	362,555	141,396
Oxalic acid	kg/year	240,535	127,965
NaOH	kg/year	393,105	157,242
Calcium hydroxide	kg/year	76,767	30,707
Total—OPEX	-	-	1,996,247

## Data Availability

Data are provided in the article.

## Abbreviations

The following abbreviations are used in this manuscript:

1.4-DCB-eq	1,4-dichlorobenzene equivalent (used in toxicity assessment)
CAPEX	Capital Expenditures
DGC	Dynamic Generation Cost
LCA	Life Cycle Assessment
LCI	Life Cycle Inventory
LCIA	Life Cycle Impact Assessment
OPEX	Operational Expenditures
REE	Rare Earth Elements
